# Negative Effects of Psychological Treatments: An Exploratory Factor Analysis of the Negative Effects Questionnaire for Monitoring and Reporting Adverse and Unwanted Events

**DOI:** 10.1371/journal.pone.0157503

**Published:** 2016-06-22

**Authors:** Alexander Rozental, Anders Kottorp, Johanna Boettcher, Gerhard Andersson, Per Carlbring

**Affiliations:** 1 Division of Clinical Psychology, Department of Psychology, Stockholm University, Stockholm, Sweden; 2 Division of Occupational Therapy, Department of Neurobiology, Care Sciences and Society, Karolinska Institutet, Stockholm, Sweden; 3 Department of Occupational Therapy, University of Illinois at Chicago, Chicago, United States of America; 4 Department of Clinical Psychology and Psychotherapy, Freie Universität Berlin, Berlin, Germany; 5 Division of Psychiatry, Department of Clinical Neuroscience, Karolinska Institutet, Stockholm, Sweden; 6 Department of Behavioural Sciences and Learning, Linköping University, Linköping, Sweden; University of Reading, UNITED KINGDOM

## Abstract

Research conducted during the last decades has provided increasing evidence for the use of psychological treatments for a number of psychiatric disorders and somatic complaints. However, by focusing only on the positive outcomes, less attention has been given to the potential of negative effects. Despite indications of deterioration and other adverse and unwanted events during treatment, little is known about their occurrence and characteristics. Hence, in order to facilitate research of negative effects, a new instrument for monitoring and reporting their incidence and impact was developed using a consensus among researchers, self-reports by patients, and a literature review: the Negative Effects Questionnaire. Participants were recruited via a smartphone-delivered self-help treatment for social anxiety disorder and through the media (*N* = 653). An exploratory factor analysis was performed, resulting in a six-factor solution with 32 items, accounting for 57.64% of the variance. The derived factors were: symptoms, quality, dependency, stigma, hopelessness, and failure. Items related to unpleasant memories, stress, and anxiety were experienced by more than one-third of the participants. Further, increased or novel symptoms, as well as lack of quality in the treatment and therapeutic relationship rendered the highest self-reported negative impact. In addition, the findings were discussed in relation to prior research and other similar instruments of adverse and unwanted events, giving credence to the items that are included. The instrument is presently available in eleven different languages and can be freely downloaded and used from www.neqscale.com.

## Introduction

Psychological treatments have the potential of alleviating mental distress and enhancing well-being for many patients suffering from psychiatric disorders and somatic complaints. Research of such methods as cognitive behavior therapy (CBT) indicate that they are effective and can have long-term benefits, both in research settings and in regular outpatient clinics [1–3]. Meanwhile, different ways of increasing access to psychological treatments have been explored, both by introducing national guidelines and recommendations to health-care providers [4–6], and by investigating the usefulness of Internet or smartphone delivered treatment interventions [7–9]. However, although promising in relation to disseminating best available care, little attention has thus far been given to the potential of negative effects of psychological treatments [10]. Most clinical trials focus on the average treatment outcome and the number of patients achieving clinical significant change, that is, attaining a positive result that fulfills a predetermined diagnostic criterion or is beyond a statistical cutoff, while ignoring the fact that some patients might also experience adverse or unwanted events [11–13]. In comparison to pharmacological research, studies involving psychological treatments seldom report the possibility of negative effects [14]. A recent review showed that only one-fifth of a large number of randomized controlled trials mentioned the occurrence of harm [15]. The situation has more or less remained the same throughout history, presumably because efforts were made to determine the efficacy of psychological treatments and establish their position in relation to medicine [16], thereby missing to examine the probability of negative effects during the treatment period. Adverse and unwanted events were, however, mentioned in an evaluation in the 1950’s regarding the Cambridge-Somerville Youth Study of delinquent adolescents, indicating that a larger proportion of those assigned to the intervention group actually were to commit more crimes than those allocated to the control group [17]. Likewise, Bergin [18] was able to provide evidence of patients deteriorating in seven different outcome studies, arguing that between five to ten percent consistently seem to deteriorate. Although obtaining critique for the difficulty of determining a causal relationship [19], that is, proving that the treatment interventions and not any other circumstances are responsible for the patients faring worse, the numbers have been confirmed in later reviews and across various treatment modalities and psychiatric disorders [20–22], suggesting that deterioration is to be expected and controlled for to reverse a negative treatment trend [23].

Deterioration is, however, far from the only negative effect that might occur during psychological treatments. Hadley and Strupp [24] were early to recognize a wide range of adverse and unwanted events, for instance, social stigma, dependency, and novel symptoms. Similarly, Mays and Franks [25], introducing the term negative outcome, argued that any type of significant decline in one or more areas of functioning during the treatment period should be regarded as negative, not just deterioration in symptomatology. Others have also implied that nonresponse, dropout, and interpersonal difficulties may be perceived as negative effects [26–28], although the prospect of establishing a cause-effect relationship is complex owing to the influence of other factors, most notably, the natural fluctuations in psychiatric disorders, the undesirable impact of everyday stressors, as well as what perspective is being used to judge whether or not a negative effect has occurred. Strupp and Hadley [29], for example, presented a tripartite model for assessing the positive as well as negative effects of psychological treatments, suggesting that the outcome will depend on the eye of the beholder: the patient, the therapist, or society at large. A specific response occurring during the treatment period, for instance, increased anxiety in an exposure exercise, might be perceived as negative by the patient, but can be expected and perhaps even regarded as beneficial by the therapist providing the treatment. Thus, even though there are reasons to assume that other types of negative effects exist in psychological treatments, determining their occurrence is complicated and warrants both theoretical and methodological considerations.

Different suggestions on how to monitor and report negative effects have, nonetheless, been put forward, and the need for more research has been emphasized [30–32]. Deterioration has, for instance, long been regarded as a relatively straightforward method for assessing the number of patients faring worse on a given outcome measure [33]. In addition, both therapist and patient administered measures have been proposed. One early attempt was the Vanderbilt Negative Indicators Scale (VNIS), a comprehensive therapist rating system to determine the occurrence of various negative effects using tape-recorded sessions [34]. The VNIS distinguished between 42 different items on five different subscales (scored 0–5), e.g., unrealistic expectations (patient personal qualities), deficiencies in therapeutic commitment (therapist personal qualities), inflexible use of therapeutic techniques (errors in technique), poor therapeutic relationship (patient-therapist interaction), and poor match (global session ratings). Albeit highly ambitious and theory driven [35], the initial evaluation only consisted of two samples of 10 and 18 patients, and the internal consistencies and interrater reliability revealed great irregularity [36]. As for their relationship with treatment outcome, errors in technique showed the strongest association, although the results seemed to vary between treatment modalities and few correlations remained significant after partialling out the effect of the other subscales. Also, with the exception for a limited number of psychodynamic psychotherapy studies [37, 38], the VNIS never became popular by researchers or therapists. Other instruments have been proposed since then, such as, the Experiences of Therapy Questionnaire (ETQ) [39]. A principal component analysis was used on data from 716 patients undergoing or having prior experiences of being in psychological treatment, revealing a rotated solution of five components explaining 53.4% of the variance. Of the original 103 items that were generated, 63 were retained (scored 1–5), e.g., “My therapist doesn’t seem to understand what I want to get out of therapy” (Item 11). The components included such areas as negative therapist (e.g., lack of empathy), pre-occupying therapy (e.g., feeling alienated), beneficial therapy (e.g., increased insight), idealization of therapist (e.g., feeling dependent on the therapist), and passive therapist (e.g., inexperienced therapist) [40]. The components were subsequently related to different sociodemographic variables, type of psychological treatment, frequency of sessions, and reasons for entering and discontinuing therapy, indicating that younger patients terminated early on because the therapist was too passive or unable to solve any problems, and that many patients believed their therapy was ineffective. Albeit using a large and heterogeneous sample in terms of psychiatric disorders and treatment modalities, the generation of items was not entirely clear and included both negative and positive effects, rather than providing an instrument that solely investigates adverse or unwanted events. Furthermore, all comparisons were made post hoc and not according to any initial hypotheses, increasing the risk of obtaining spurious findings. Linden [41], on the other hand, presented a different approach to examining negative effects, the Unwanted to Adverse Treatment Reaction (UE-ATR) checklist, a therapist-administered instrument for assessing a wide range of potential adverse and unwanted effects, for instance, lack of clear treatment results, prolongation of treatment, and non-compliance of the patient. The therapist is also supposed to determine how the negative effects were linked to the psychological treatment using a five-step scale ranging from unrelated to related, and an evaluation of their severity level, e.g., mild, moderate, and severe. Conceptually, the UE-ATR resembles the VNIS in that the negative effects can involve different areas of life, not only deterioration of symptomatology, and that the relationship with treatment is not always clear. However, as stated by Linden and Schermuly-Haupt [30], the instrument is more of a tool for improving the therapist’s ability to detect negative effects than a scale with distinguishable psychometric properties, although it has been used in at least one clinical trial [42]. As for other instruments, the Inventory for the Assessment of Negative Effects of Psychotherapy (INEP) has also been put forward [43]. After performing a literature review and consulting psychotherapy researchers, 120 items were generated (scored on a three-step scale regarding change or a four-step scale in terms of agreement), such as, “I feel addicted to my therapist” (Item 10). Of these, 52 items were selected and distributed to 195 patients that had undergone psychological treatment and who were recruited via advertisements. Using a principal component analysis and a confirmatory factor analysis, the results yielded a rotated solution of five or seven components/factors, depending on the type of analysis; intrapersonal changes, intimate relationship, stigmatization, emotions, workplace, therapeutic malpractice, and family and friends, accounting for 46.7 or 55.8% of the variance (the final version consists of 21 items). Interestingly, the results indicated that more patients in behavioral than psychodynamic or nondirective therapy felt forced by their therapist to implement certain interventions, while patients in nondirective therapy had longer periods of depression after the treatment period, and patients in psychodynamic therapy more frequently felt offended by their therapist. Although carefully developed and providing some useful recommendations, most notably, asking the patient to differentiate between negative effects of their treatment and other circumstances, the INEP is difficult to score and assess in relation to treatment outcome as it does not include a clear and coherent scale. Further, several items could be criticized on theoretical grounds, for instance, “I have trouble finding insurance or am anxious to apply for new insurances” (Item 8), as it might not always be applicable in different contexts. Also, a large number of items seem to convey malpractice issues, such as, “My therapist attacked me physically” (Item 19), and not negative effects of properly performed psychological treatments. Although they most certainly will have a negative impact, it could be argued that malpractice issues are related to the unethical behavior of a therapist rather than a feature of the treatment interventions [11].

Hence, in order to address some of the shortcomings that have been mentioned, a new instrument for assessing negative effects of psychological treatments was developed: the Negative Effects Questionnaire (NEQ). Items were generated by consulting a number or researchers [32], distributing open-ended questions [42], analyzing patient responses using qualitative method [44], and a comprehensive literature review. The purpose of this process was to present an instrument that is based on both theoretical considerations and empirical findings, with items being systematically derived, reasonable to expect, and comprehensible for the patient. The overall purpose of the current study is to determine the validity and factor structure of the instrument, and to examine what items should be retained in a final version. This is believed to result in an instrument that is accessible and easier to administer by researchers and therapists, which might aid the investigation of negative effects in a variety of different psychological treatments and to explore their relationship with treatment outcome. Providing an instrument that can identify adverse and unwanted events during the treatment period may also help therapists identify patients at risk of faring worse, and to offer other treatment interventions as a way of reversing a negative treatment trend.

## Methods

### Item design

Items were carefully generated using a consensus statement regarding the monitoring and reporting of negative effects [32], findings from a treatment outcome study of patients with social anxiety disorder that probed for adverse and unwanted events [42], the results of a qualitative content analysis of the responses from four different clinical trials [44], and a literature review of books and published articles on negative effects. This is in line with the recommendations by Cronbach and Meehl [45], advising researchers to articulate the theoretical concept of an instrument before developing and testing it empirically in order to increase content validity. Also, instead of restricting the number of items to be included in a final version, the concept of overinclusiveness was adapted, that is, embracing more items than necessary to aid the statistical analyses necessary for detecting those that are related to the underlying construct(s) [46]. Subsequently, 60 items were generated, characterized by interpersonal issues, problems with therapeutic relationship, deterioration, novel symptoms, stigma, dependency, hopelessness, difficulties understanding the treatment content, as well as problems implementing the treatment interventions. An additional open-ended question was also included for the investigation of negative effects that might have been experienced but were not listed, i.e., “Describe in your own words whether there were any other negative incidents or effects, and what characterized them”. Further, in order to assess the readability and understanding of the items, cognitive interviews were conducted on five individuals unrelated to the current study and without any prior knowledge of negative effects or psychological treatments, i.e., encouraging them to read the items out load and speak freely of whatever comes to mind [47]. Cognitive interviews are often suggested as a way of pretesting an instrument so that irrelevant or difficult items can be revised and to increase its validity [48]. In relation to the proposed items, several minor changes were made, e.g., rephrasing or clarifying certain expressions. In addition, the instrument included general information about the possibility of experiencing negative effects, and was comprised of three separate parts; 1) “Did you experience this?” (yes/no) 2) “If yes–here is how negatively it affected me” (not at all, slightly, moderately, very, and extremely), and 3) “Probably caused by” (the treatment I received/other circumstances). The instrument is scored 0–4 and contains no reversed items as this may introduce errors or artifacts in the responses [49].

### Data collection

The instrument was distributed via the Internet using an interface for administering surveys and self-report measures, Limesurvey (www.limesurvey.org). Participants were recruited via two different means in order to include a diverse and heterogeneous sample: patients undergoing a smartphone-delivered self-help treatment for social anxiety disorder based on CBT (*N* = 189) [50], and individuals responding to an article on negative effects of psychological treatments featured in the largest morning newspaper in Sweden as well as a Swedish public radio show on science with the same topic, (*N* = 464), yielding a total sample size of 653. As for the treatment group, patients were instructed to complete the instrument on negative effects while responding to the outcome measures at the post treatment assessment, resulting in a response rate of 90.4%. In terms of the media group, information on negative effects and the purpose of the current study was presented on a website specifically created for the purpose of the current study (www.psykoterapiforskning.se), where the individuals were instructed to fill out the instrument and information on sociodemographics, rendering a response rate of 49.4% (defined as those who entered the website and completed the instrument). Inclusion criteria for the treatment group, that is, to be included in the clinical trial, were; above 30 points on the Liebowitz Social Anxiety Scale–Self-Report [51], social anxiety disorder according to The Mini-International Neuropsychiatric Interview (MINI) [52], access to an IPhone, at least 18 years of age, and being a Swedish resident. Suicidality, ongoing psychological treatment, or a recent commencement or alteration of any psychotropic medication were all reasons for exclusion from the clinical trial. With regard to the media group, inclusion criteria comprised only of having undergone or being in psychological treatment sometime during the last two years. None of the two groups received any monetary compensation to complete the instrument.

### Statistical analysis

All data was assembled and organized in one main dataset, and the statistical analyses were performed on IBM SPSS Statistics, version 22. As the purpose of the current study was to present an instrument for assessing negative effects of psychological treatments, only items that were attributable to treatment by the participants were analyzed. In order to determine the validity and factor structure of the instrument, an exploratory factor analysis (EFA) was conducted using principal axis factoring. This method is suitable for assessing theoretically interesting latent constructs rather than to test a specific hypothesis [53], corresponding to the purpose of the current study. Also, for an EFA to be appropriate, the level of measurement must be considered to be interval, or, at least quasi-interval, which could be assumed for the data that were collected [54]. In comparison to other methods for investigating the underlying dimensions of an instrument, such as, principal component analysis, an EFA also accounts for measurement error, argued to result in more realistic assumptions [55]. As for the rotated solution used for extracting the number of factors, an oblique rotation was implemented using direct oblimin with delta set to zero and the number of iterations set to 40. As discussed by Browne [56], an oblique rotation permits factors to be correlated, which orthogonal rotation does not, and is thus more representative of social science data where it is reasonable to assume that different factors in the same instrument will in fact correlate to some degree. Additional analyses implemented for considering the appropriateness of EFA were the Kaiser-Meyer-Olkin (KMO) measure of sampling adequacy, assessing the potential for finding distinct and reliable factors, the Bartlett’s Test of Sphericity, which indicates if the correlations between items are significantly different from zero, as well as the Determinant, checking for a reasonable level of correlations. In addition, item-item correlations < .30 or >.90 were considered to see if items measure the same underlying construct and to investigate the risk of multicollinearity. In order to establish the validity of the extracted factor solution, several methods were used. Eigenvalues greater than one, the Kaiser criterion, was only utilized as a preliminary analysis, given that it has been found to result in both over- and underfactoring [57]. The scree test was then implemented to visually inspect the number of factors that precedes the last major drop in eigenvalues [58], although it needs to be validated by other means as it is deemed a highly subjective procedure [59]. Hence, parallel analysis was performed, i.e., comparing the obtained factor solution with one derived from data that is produced at random with the same number of cases and variables, meaning that the correct number of factors should equal to eigenvalues higher than those that are randomly generated [60]. As SPSS does not perform parallel analysis, syntax from O’Connor [61] was used. Moreover, to examine the validity of the factor solution across samples, a stability analysis was conducted by making SPSS select half of the cases at random and then retesting the factor solution [53], with similar results indicating if its relatively stable. The interpretability of the factors was also checked to see if it was reasonable and fits well with prior theoretical assumptions and empirical findings [62].

### Ethical considerations

All data included in the current study were manually imputed by the participants and assigned an auto generated identification code, i.e., 1234abcd, allowing complete anonymity. As for the treatment group, ethical approval was obtained by the Regional Ethical Board in Stockhom, Sweden (Dnr: 2014/680-31/3), and written informed consent was collected by letter at the pre treatment assessment. The consent form included information regarding the clinical trial, how to contact the principal investigator, data management and confidentiality, and the right to obtain a copy of one’s personal record in accordance with the Swedish Personal Data Act. With regard to the media group, information about the authors as well as the current study was provided, and a written informed consent with the same details as above was submitted digitally before responding to the instrument. Moreover, the results are only presented on group level, and great consideration was made in order not to disclose the identity of a specific participant.

## Results

### Participants

A total sample of 653 participants was included in the current study, with a majority being women (76.6%), in their late thirties, and in a relationship (60%). A large proportion had at least a university degree (62%) and were either employed (52.7%) or students (25.1%). In terms of the reason for receiving psychological treatment according to the participants themselves, anxiety disorders were most prevalent (48.4%), compared to mixed anxiety/depression (14.1%), depression (10.1%), and other psychiatric disorders or psychological problems (27.4%). As for the therapeutic orientation the participants believed they had received, cognitive/behavioral was predominant (61.3%), which includes several different modalities, e.g., schema therapy, cognitive therapy, as well as acceptance and commitment therapy, followed by psychodynamic psychotherapy (17.2%). Prior or ongoing psychotropic medication was also relatively common (38.3%). See Table 1 for an overview of the participants, divided by means of recruitment.

**Table 1:** Sociodemographic characteristics of participants divided by means of recruitment.

	Treatment group (*n* = 189)	Media group (*n* = 464)	Total sample (*n* = 653)
Gender: *n* (% female)	146 (77.2)	354 (76.3)	500 (76.6)
Age (years): *M* (*SD*)	35.3 (12.5)	38.0 (12.3)	37.2 (12.4)
Civil status: *n* (%)			
Single	64 (33.9)	194 (41.8)	258 (39.5)
Relationship	122 (64.6)	270 (58.2)	392 (60)
Other	3 (1.6)	n.a. ^c^	3 (0.5)
Children: *n* (% yes)	95 (50.3)	n.a. ^c^	95 (14.5)
Cohabitant: *n* (% yes)	134 (70.9)	n.a. ^c^	134 (20.5)
Highest educational level: *n* (%)			
Elementary school	10 (5.3)	18 (3.9)	28 (4.3)
High school/college	73 (38.6)	147 (31.7)	220 (33.7)
University	104 (55.0)	287 (61.9)	391 (59.9)
Postgraduate	2 (1.1)	12 (2.6)	14 (2.1)
Employment: *n* (%)			
Unemployed	14 (7.4)	28 (6.0)	42 (6.4)
Student	45 (23.8)	119 (25.6)	164 (25.1)
Employed	119 (63.0)	225 (48.5)	344 (52.7)
Parental leave	4 (2.1)	11 (2.4)	15 (2.3)
Retired	4 (2.1)	22 (4.7)	26 (4.0)
Sick-leave	3 (1.6)	59 (12.7)	62 (9.5)
Primary diagnosis: *n* (%)			
Anxiety disorder	189 (100)	127 (27.4)	316 (48.4)
Anxiety and depression	n.a. ^a^	92 (19.8)	92 (14.1)
Depression	n.a. ^a^	66 (14.2)	66 (10.1)
Other	n.a. ^a^	179 (38.6)	179 (27.4)
Therapeutic orientation			
Cognitive/behavioral	189 (100)	211 (45.5)	400 (61.3)
Psychodynamic	n.a. ^b^	112 (24.0)	112 (17.2)
Integrative	n.a. ^b^	30 (6.5)	30 (4.6)
Unclear	n.a. ^b^	82 (17.7)	82 (12.5)
Other	n.a. ^b^	29 (6.3)	29 (4.4)
Prior psychological treatment *n* (% yes)	79 (41.8)	n.a. ^d^	79 (12.1)
Prior or ongoing psychotropic medication *n* (% yes)	54 (28.6)	196 (42.2)	250 (38.3)

n.a. = not applicable

^a^ Not applicable as diagnosis

^b^ Not applicable as treatment orientation

^c^ Not applicable as response alternatives

^d^ Not applicable as prior or ongoing psychological treatment was an inclusion criterion

### Principal axis factoring

The preliminary assessment revealed a KMO of .94 and that the Bartlett’s Test of Sphericity was significant. Also, the Determinant indicated a reasonable level of correlations, suggesting that the data was suitable for performing an EFA. None of the off-diagonal items had correlations of >.90, suggesting no risk of multicollinearity. However, fourteen items had a large number of correlations of < .30 and were therefore subject for further investigation. Furthermore, four items specifically related to Internet-based psychological treatments, e.g., “I wasn’t satisfied by the user interface in which the treatment was being delivered” (Item 58), only consisted of correlations below the threshold and were deemed susceptible for removal. The communality estimates of the extracted factor solution, which reflects each item’s variance explained by all of the factors in the model, resulted in an average of .52, recommending the use of the scree test as an aid to the Kaiser criterion to determine the number of factors to retain. In terms of the former, a three-factor solution seemed reasonable, but using the latter, five factors had an eigenvalue greater than one, with an additional two factors being >.90, explaining a variance of 45.50%. Albeit resulting in two factor solutions, retaining seven factors was regarded most appropriate and was used for further examination.

A closer inspection of the extracted factor solution indicated that two items could be removed as the correlations were too small or because they would enhance the internal consistency if replaced. Moreover, the seventh factor was only comprised of items that conveyed negative effects of Internet-based psychological treatments, which previously had been found to be unrelated to the underlying construct(s). Therefore, a six factor solution seemed more sensible to maintain, whereby an EFA was performed using only six factors and with the problematic items having been removed. The results indicated that four factors were above the Kaiser criterion, one was >.90, and one resulted in an eigenvalue of .68, accounting for 57.64% of the variance. Although the last factor was well below the threshold, it was considered appropriate for retention due to theoretical reasons, that is, reflecting the experience of failure during psychological treatment. For a full overview of the specific items, the six-factor solution, and the correlations between each item and their respective factor can be found in Table 2.

**Table 2:** Principal axis factoring for a six factor solution using oblique rotation.

Item	Factor 1: Symptoms	Factor 2: Quality	Factor 3: Dependency	Factor 4: Stigma	Factor 5: Hopelessness	Factor 6: Failure
1. I had more problems with my sleep	.572					
2. I felt like I was under more stress	.534					
3. I experienced more anxiety	.700					
4. I felt more worried	.554					
5. I felt more dejected	.625					
6. I experienced more hopelessness					.373	
7. I experienced lower self-esteem						.677
8. I lost faith in myself						.708
9. I felt sadder	.616					
10. I felt less competent						.555
11. I experienced more unpleasant feelings	.703					
12. I felt that the issue I was looking for help with got worse	.431					
13. Unpleasant memories resurfaced	.692					
14. I became afraid that other people would find out about my treatment				.738		
15. I got thoughts that it would be better if I did not exist anymore and that I should take my own life	.487					
16. I started feeling ashamed in front of other people because I was having treatment				.771		
17. I stopped thinking that things could get better					-.798	
18. I started thinking that the issue I was seeking help for could not be made any better					-.719	
19. I stopped thinking help was possible					-.626	
20. I think that I have developed a dependency on my treatment			.820			
21. I think that I have developed a dependency on my therapist			.819			
22. I did not always understand my treatment		-.516				
23. I did not always understand my therapist		-.634				
24. I did not have confidence in my treatment		-.849				
25. I did not have confidence in my therapist		-.844				
26. I felt that the treatment did not produce any results		-.605				
27. I felt that my expectations for the treatment were not fulfilled		-.640				
28. I felt that my expectations for the therapist were not fulfilled		-.784				
29. I felt that the quality of the treatment was poor		-.793				
30. I felt that the treatment did not suit me		-.649				
31. I felt that I did not form a closer relationship with my therapist		-.592				
32. I felt that the treatment was not motivating		-.615				

**Fig 1:**
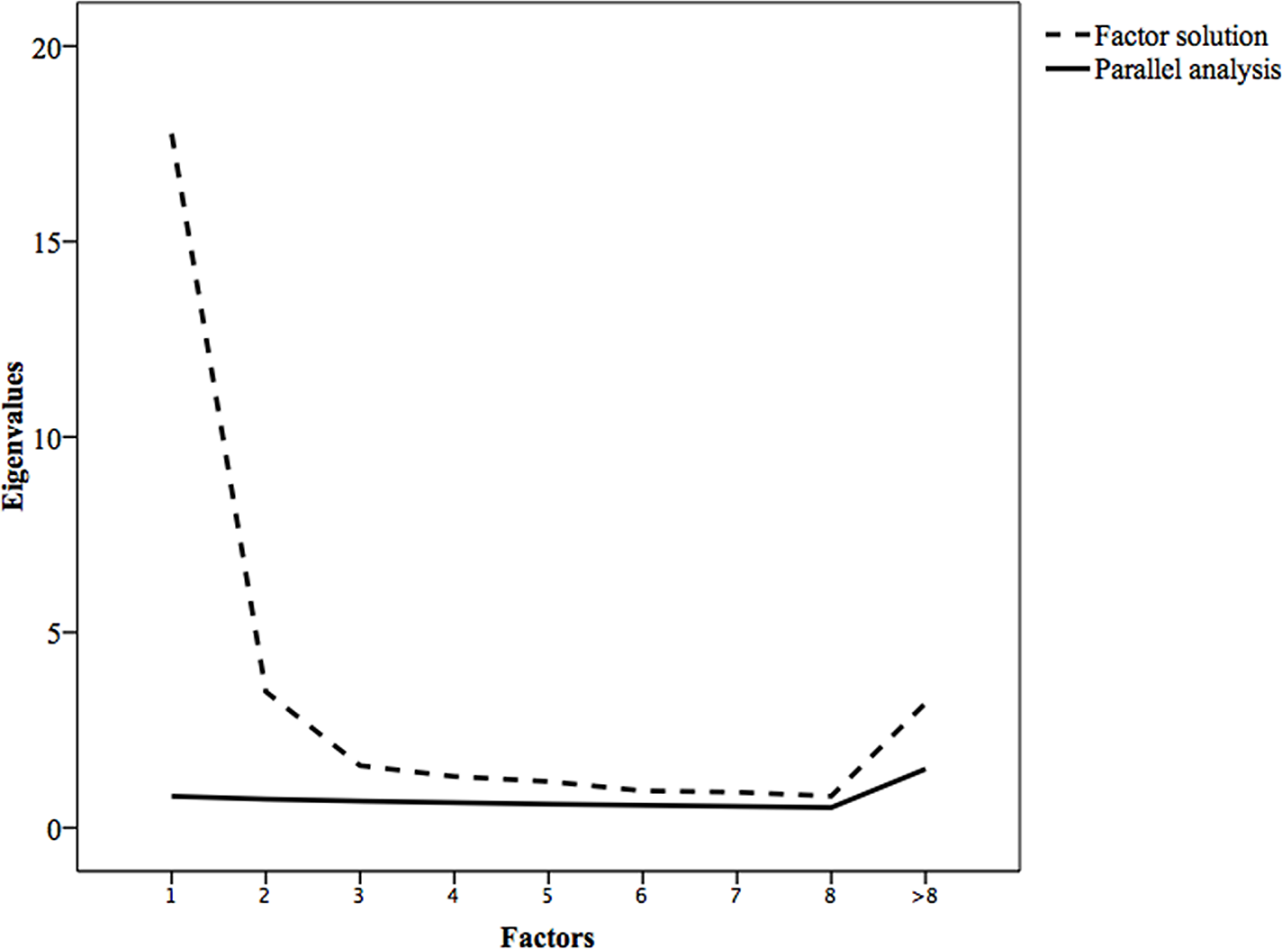
Parallel analysis of the factor solution.

In order to validate the six-factor solution, a parallel analysis was performed using a permutation test of 1000 iterations with the same number of cases and variables as the original dataset. That is, similar to bootstrapping procedures, a total of 1000 random datasets were produced, and an average eigenvalue and 95% Confidence Interval (CI) was reported for each factor. Both according to the scree test and a comparison between the eigenvalues obtained in the six-factor solution and the parallel analysis indicated that the original factor solution was reasonable to retain. Hence, none of the six factors were below the mean eigenvalues or 95% CI of the random of the randomly generated datasets. For a visual inspection please refer to Fig 1.

Further, as a measure of validity across samples, a stability analysis was conducted by making SPSS randomly select half of the cases and retesting the factor solution. The results indicated that the same six-factor solution could be retained, albeit with slightly different eigenvalues, implying stability. A review of the stability analysis can be obtained in Table 3.

**Table 3:** Stability analysis of the six-factor solution using a randomly selected sample.

		Original sample (*N* = 653)			Random sample (*N* = 326)		
		Eigen value	Variance %	Cumulative %	Eigen value	Variance %	Cumulative %
1	Symptoms	11.71	36.58	36.58	12.45	38.91	38.91
2	Quality	2.79	8.71	45.29	2.85	8.90	47.81
3	Dependency	1.32	4.13	49.42	1.50	4.68	52.49
4	Stigma	1.01	3.16	52.59	1.10	3.43	55.92
5	Hopelessness	0.94	2.94	55.53	0.93	2.89	58.81
6	Failure	0.68	2.11	57.64	0.59	1.84	60.65

### Factor solution

The final factor solution consisted of six factors, which included 32 items. A closer inspection of the results revealed one factor related to “symptoms”, e.g., “I felt more worried” (Item 4), with ten items reflecting different types of symptomatology, e.g., stress and anxiety. Another factor was linked to “quality”, e.g., “I did not always understand my treatment” (Item 23), with eleven items characterized by deficiencies in the psychological treatment, e.g., difficulty understanding the treatment content. A third factor was associated with “dependency”, e.g., “I think that I have developed a dependency on my treatment” (Item 20), with two items indicative of becoming overly reliant on the treatment or therapist. A fourth factor was related to “stigma”, e.g., “I became afraid that other people would find out about my treatment” (Item 14), with two items reflecting the fear of being perceived negatively by others because of undergoing treatment. A fifth factor was characterized by “hopelessness”, e.g., “I started thinking that the issue I was seeking help for could not be made any better” (Item 18), with four items distinguished by a lack of hope. Lastly, a sixth factor was linked to “failure”, e.g., “I lost faith in myself” (Item 8), with three items connected to feelings of incompetence and lowered self-esteem.

Table 4 contains the means, standard deviations, internal consistencies, and correlations among the factors. With regard to the full instrument, α was .95, while it ranged from .72-.93 for the specific factors: lowest for stigma, and highest for quality. The largest correlations were obtained between quality and hopelessness, *r* = .55, symptoms and failure, *r* = .50, and hopelessness and failure, *r* = -.49.

**Table 4:** Means, standard deviations, internal consistencies, and correlates among the obtained factors.

					Factor					
		*M*	*SD*	α	1	2	3	4	5	6
1	Symptoms	21.43	14.63	.90		-.40	.26	.28	-.45	.50
2	Quality	30.82	5.83	.93	-.40		-.09	-.18	.55	-.40
3	Dependency	4.21	2.74	.82	.26	-.09		.18	-.12	.16
4	Stigma	3.47	7.16	.72	.28	-.18	.18		-.20	.19
5	Hopelessness	7.19	3.84	.87	-.45	.55	-.12	-.20		-.49
6	Failure	6.84	4.34	.84	.50	-.40	.16	.19	-.49	
	Full instrument	20.38	26.10	.95						

In terms of the items that were most frequently endorsed as occurring during treatment, participants experienced; “Unpleasant memories resurfaced” (Item 13), 38.4%, “I felt like I was under more stress” (Item 2), 37.7%, and “I experienced more anxiety” (Item 3), 37.2%. Likewise, the items that had the highest self-rated negative impact were; “I felt that the quality of the treatment was poor” (Item 29), 2.81 (*SD* = 1.10), “I felt that the issue I was looking for help with got worse” (Item 12), 2.68 (*SD* = 1.44), and “Unpleasant memories resurfaced” (Item 13), 2.62 (*SD* = 1.19). A full review of the items can be obtained in Table 5.

**Table 5:** Items, number of responses, mean level of negative impact, and standard deviations.

Item	Responses n (%)	*M*	*SD*
1. I had more problems with my sleep	135 (20.7)	1.70	1.72
2. I felt like I was under more stress	246 (37.7)	1.84	1.62
3. I experienced more anxiety	243 (37.2)	2.09	1.54
4. I felt more worried	191 (29.2)	2.04	1.58
5. I felt more dejected	194 (29.7)	1.88	1.61
6. I experienced more hopelessness	140 (21.4)	2.15	1.55
7. I experienced lower self-esteem	120 (18.4)	2.18	1.51
8. I lost faith in myself	115 (17.6)	2.11	1.58
9. I felt sadder	229 (35.1)	1.99	1.46
10. I felt less competent	117 (17.9)	2.16	1.44
11. I experienced more unpleasant feelings	199 (30.5)	2.35	1.38
12. I felt that the issue I was looking for help with got worse	112 (17.2)	2.68	1.44
13. Unpleasant memories resurfaced	251 (38.4)	2.62	1.19
14. I became afraid that other people would find out about my treatment	88 (13.5)	1.63	1.38
15. I got thoughts that it would be better if I did not exist anymore and that I should take my own life	97 (14.9)	1.11	1.55
16. I started feeling ashamed in front of other people because I was having treatment	57 (8.7)	1.65	1.58
17. I stopped thinking that things could get better	126 (19.3)	2.21	1.52
18. I started thinking that the issue I was seeking help for could not be made any better	165 (25.3)	2.21	1.55
19. I stopped thinking help was possible	122 (18.7)	2.25	1.62
20. I think that I have developed a dependency on my treatment	74 (11.3)	2.05	1.26
21. I think that I have developed a dependency on my therapist	68 (10.4)	2.03	1.37
22. I did not always understand my treatment	207 (31.7)	2.24	1.09
23. I did not always understand my therapist	166 (25.4)	2.19	1.25
24. I did not have confidence in my treatment	129 (19.8)	2.43	1.21
25. I did not have confidence in my therapist	114 (17.5)	2.50	1.22
26. I felt that the treatment did not produce any results	169 (25.4)	2.58	1.43
27. I felt that my expectations for the treatment were not fulfilled	219 (33.5)	2.36	1.37
28. I felt that my expectations for the therapist were not fulfilled	138 (21.1)	2.60	1.22
29. I felt that the quality of the treatment was poor	113 (17.3)	2.81	1.10
30. I felt that the treatment did not suit me	159 (24.4)	2.49	1.33
31. I felt that I did not form a closer relationship with my therapist	182 (27.9)	1.95	1.43
32. I felt that the treatment was not motivating	111 (17.0)	2.59	1.18

## Discussion

The current study evaluated a new instrument for assessing different types of negative effects of psychological treatments; the NEQ. Items were generated using consensus among researchers, experiences by patients having undergone treatment, and a literature review. The instrument was subsequently administered to patients having received a smartphone-delivered self-help treatment for social anxiety disorder and individuals recruited via two media outlets, having received or were currently receiving treatment. An investigation using EFA revealed a six-factor solution with 32 items, defined as: symptoms, quality, dependency, stigma, hopelessness, and failure. Both a parallel analysis and a stability analysis suggested that the obtained factor solution could be valid and stable across samples, with an excellent internal consistency for the full instrument and acceptable to excellent α for the specific factors. The results are in line with prior theoretical assumptions and empirical findings, giving some credibility to the factors that were retained. Symptoms, that is, deterioration and distress unrelated to the condition for which the patient has sought help, have frequently been discussed in the literature of negative effects [24, 26, 30]. Research suggests that 5–10% of all patients fare worse during the treatment period, indicating that deterioration is not particularly uncommon [63]. Furthermore, evidence from a clinical trial of obsessive-compulsive disorder indicates that 29% of the patients experienced novel symptoms [64], suggesting that other types of adverse and unwanted events may occur. Anxiety, worry, and suicidality are also included in some of the items of the INEP [43], implying that various symptoms are to be expected in different treatment settings. However, these types of negative effects might not be enduring, and, in the case of increased symptomatology during certain interventions, perhaps even expected. Nonetheless, given their occurrence, the results from the current study recommends the monitoring of symptoms by using the NEQ in case they affect the patient’s motivation and adherence. Likewise, the perceived quality of the treatment and relationship with the therapist are reasonable to influence well-being and the patient’s motivation to change, meaning that a lack of confidence in either one may have a negative impact. This is evidenced by the large correlation between quality and hopelessness, suggesting that it could perhaps affect the patient’s hope of attaining some improvement. Research has revealed that expectations, specific techniques, and common factors, e.g., patient and therapist variables, may influence treatment outcome [65]. In addition, several studies on therapist effects have revealed that some could potentially be harmful for the patient, inducing more deterioration in comparison to their colleagues [66], and interpersonal issues in treatment have been found to be detrimental for some patients [67]. Similarly, difficulties understanding the treatment or purpose of specific interventions could be regarded as negative by the patient, presumably affecting both expectations and self-esteem. Items reflecting deficiencies and lack of credibility of the treatment and therapist are also included in both the ETQ and INEP [39, 43], making it sensible to expect negative effects due to lack of quality. With regard to dependency, the empirical findings are less clear. Patients becoming overly reliant on their treatment or therapist have frequently been mentioned as a possible adverse and unwanted event [13, 24, 41], but the evidence has been missing. In reviewing the results from questionnaires, focus groups, and written complaints, a recent study indicated that 17.9% of the surveyed patients felt more dependent and isolated by undergoing treatment [68]. Both the ETQ and INEP also contain items that are related to becoming addicted to treatment or the therapist [39, 43]. Hence, it could be argued that dependency may occur and is problematic if it prevents the patient from becoming more self-reliant. However, the idea of dependency as being detrimental is controversial given that it is contingent on both perspective and theoretical standpoint. Dependency may be regarded as negative by significant others, but not necessarily by the patient [29]. Also, dependency could be seen as beneficial with regard to establishing a therapeutic relationship, but adverse and unwanted if it hinders the patient from ending treatment and becoming an active agent [69]. Determining the issue of dependency directly, as in using the NEQ, could shed some more light on this matter and warrants further research. In terms of stigma, little is currently known about its occurrence, characteristics, and potential impact. Linden and Schermuly-Haupt [30] discuss it as a possible area for assessing negative effects. Being afraid that others might find out about one’s treatment is also mentioned in the INEP [43]. Given the fact that much have been written about stigma and its interference with mental health care [70–72], there is reason to assume that the idea of being negatively perceived by others for having a psychiatric disorder or seeking help could become a problem in treatment. However, whether stigma should be perceived as a negative effect attributable to treatment or other circumstances, e.g., social or cultural context, remains to be seen. As for hopelessness, the relationship is much clearer. Lack of improvement and not believing that things can get better are assumed to be particularly harmful in treatment [28], and could be associated with increased hopelessness [73]. Hopelessness is, in turn, connected to several negative outcomes, most notably, depression and suicidality [74], thus being of great importance to examine during treatment. Hopelessness is included in instruments of depression, e.g., the Beck Depression Inventory [75], “I feel the future is hopeless and that things cannot improve” (Item 2), and is vaguely touched upon in the ETQ [39], i.e., referring to non-improvement. Assessing it more directly by using the NEQ should therefore be of great value, particularly given its relationship with more severe adverse events. Lastly, failure has been found to be linked to increased stress and decreased well-being [76], especially if accompanied by an external as opposed to internal attributional style [77], making it difficult to adequately cope with setbacks [78]. Experiencing difficulties during treatment, as well as not improving, could be presumed to be negative for the patient, resulting in lower self-esteem and competency. Correlations between the factors give some support for this idea, as both symptoms and hopelessness revealed moderate to large associations with failure. The ETQ mentions failure in one of its items [39], but only in terms of the therapist making the patient feel incompetent. Feelings of failure could be particularly damaging if it leads to drop out and prevents the patient from seeking treatment in the future, suggesting that the NEQ might be useful for monitoring this issue more closely.

As to the items that were most frequently endorsed as occurring during treatment, unpleasant memories, stress, and anxiety were each experienced by more than one-third of the participants in the current study. Other items associated with symptoms were also common, indicating that adverse and unwanted events linked to novel and increased symptomatology in treatment should be reasonable to expect. This is further evidence by the fact that this factor alone accounted for 36.58% of the variance in the EFA. In addition, five items related to the quality of the treatment were each endorsed by at least one-quarter of the participants, suggesting that this too might constitute a recurrent type of negative effect. Items related to the same two factors also contributed with the highest self-rated negative impact, implying that perceiving the treatment or therapeutic relationship as deficient, or experiencing different types of symptoms could be harmful for the patient. Thus, in order to prevent negative effects from occurring, different actions might be necessary to ensure a good treatment-patient fit, i.e., the right type of treatment for a particular patient, instilling confidence, as well as dealing with the patient’s expectations of treatment and bond with the therapist. Additionally, monitoring and managing symptoms by using the NEQ would also be important [23], especially given the fact that many therapists are unaware or have not received adequate training of negative effects in treatment [79].

The current study indicates that negative effects of psychological treatments seem to occur and can be assessed using the NEQ, revealing several distinct but interrelated factors. Several limitations, however, need to be considered in reviewing the results. First, distribution of the instrument was made to patients at post treatment assessment or to individuals remembering their treatment retrospectively, with few participants presently being in treatment. Thus, there is a strong risk of recall effects exerting an influence, e.g., forgetting some adverse and unwanted events that have occurred, or only recognizing negative effects that happened early on or very late in treatment, i.e., primacy-recency effects [48]. Administering the NEQ on more than one occasion, e.g., mid-assessment, could perhaps prevent some of this problem and is therefore recommended in future studies. Although, recurrently probing for negative effects may pose a risk of inadvertently inducing adverse and unwanted events, i.e., making the patient more aware of certain incidents, which also needs to be recognized. Moreover, it may be important to explore whether the negative effects that are reported differ between those currently undergoing psychological treatment and those that have recently ended it, particularly because it could be affected by the treatment interventions they are receiving. This is also true for different treatment modalities, as it could be argued that the participants in the treatment group experienced negative effects that are very specific for a smartphone-delivered self-help treatment for social anxiety disorder. The inclusion of the media group, which was more heterogeneous in nature, may have prevented some of this problem, but further research should be conducted with more diverse samples in mind. Second, providing a list of negative effects is regarded as an aid for the participants in order to recollect adverse and unwanted events that might have been experienced during treatment. However, such alternatives could also potentially affect the responses made by the participant, that is, choosing among negative effects that may not otherwise have been considered [80]. Given that the items included in the NEQ were partly developed using the results from open-ended questions, the alternatives should nevertheless still reflect adverse and unwanted events that are reasonable to assume among the participants. Third, with regard to the sensitive issue surrounding negative effects of psychological treatments, an instrument probing for adverse and unwanted events is probably prone to produce social desirability or induce other types of biases. Krosnick [48] provides a lengthy discussion on this issue, suggesting that norms, cohesion, and personal characteristics influence a participant’s ability to respond truthfully and validly. It could be argued that patients that are satisfied with the outcome of their treatment choose not to respond because of gratitude toward the researcher or therapist. Similarly, patients that are displeased with their treatment or therapist may decline to answer, or, alternatively, exaggerate the responses in order to convey their discontent. This is particularly relevant in relation to the media group, where the participants were recruited on the grounds of having experienced negative effects, making it plausible that only those who were unhappy about their psychological treatments responded, creating selection bias. Hence, future investigations should aim to replicate the findings in the current study by distributing the NEQ to random samples, for instance, at different outpatient clinics. Likewise, despite a low dropout rate from the treatment group (9.6%), it is possible that those who did not complete the post treatment assessment, including the NEQ, may have been those who experienced deterioration, nonresponse, or adverse and unwanted events to a greater degree. Thus, the findings in the current study may have missed negative effects that were perceived but just not reported. Again, distributing the NEQ not only at post treatment assessment should avoid some of this shortcoming, as would follow-up interviews on those who choose not to continue with the treatment program. Fourth, administering an instrument that includes 60 items pose a risk of introducing a cognitive load on the participants, especially if used in adjunct to other measures. This could have affected the validity of the responses as research indicates that participants often try to preserve mental resources when filling out different questionnaires, compromising the quality for more arbitrarily chosen answers [80]. In relation to the individuals in the media group this may not have been an issue, but for the patients in the treatment group the instrument developed for the current study was one of seven outcome measures to be completed. Thus, for future studies, the problem of cognitive load needs to be considered. The NEQ now consists of 32 items and should avoid some of this problem, but the administration of the instrument on a separate occasion is nonetheless recommended. Fifth, albeit the current study has provided some evidence of negative effects of psychological treatments, the association between its occurrence and implications for outcome is still unclear. Adverse and unwanted events that arise during treatment might be a transient phenomenon related to either the natural fluctuations in psychiatric disorders or treatment interventions that are negatively experienced by the patient, but helpful in the long-run. Alternatively, such negative effects may have an impact that prevents the patient from benefitting from treatment, resulting in deterioration, hopelessness, and a sense of failure. To investigate this issue, the NEQ therefore needs to be accompanied by other outcome measures. By collecting data from several time points throughout treatment and relating it to more objective results, both at post treatment assessment and follow-up, it should be possible to determine what type of impact adverse and unwanted events actually have for the patient. Sixth, even though there exist several methods for validating a factor solution from an EFA, the findings are still to some extent a result of making subjective choices [53]. Relying solely on the Kaiser criterion or scree test provide a relatively clear criterion for obtaining the factor solution, such as, using eigenvalues greater than one as a cutoff, but risk missing factors that are theoretically relevant for the underlying construct(s) [54]. Likewise, such methods often lead to over- or underfactoring and is thus not regarded as the only mean for determining the number of factors to retain [57]. In the current study, a six-factor solution seemed most reasonable, particularly as it fits well with prior theoretical assumptions and empirical findings, which is one way of validating the results [62]. A parallel analysis and a stability analysis also provided some support for the findings, but such methods also have a number of limitations [53]. Most notably, factors that are randomly generated still have to be compared to a factor solution that is subjectively chosen, and the selection of a random number of cases to retest the factors are still derived from the same sample. Thus, it should be noted that replications are needed to fully ascertain if the obtained factor solution is truly valid and stable across samples. This would, however, warrant recruiting patients and individuals from additional settings, and to implement alternative statistical methods, such as Rasch-analysis, which has some benefits in investigating data where the level of measurement can be assumed to be quasi-interval [81]. Lastly, using EFA to determine theoretically interesting latent constructs does not imply that the items that were not retained are inapt, only that they did not fit the uni- or multidimensionality of the final factor solution. Hence, some of the items that were originally generated may still be clinically relevant, and the open-ended question included in the instrument may in the future reveal other items that are of interest.

## Conclusions

The current study tested an instrument for measuring adverse and unwanted events of psychological treatments, the NEQ, and was evaluated using EFA. The results revealed a six-factor solution with 32 items, defined as: symptoms, quality, dependency, stigma, hopelessness, and failure, accounting for 57.64% of the variance. Unpleasant memories, stress, and anxiety were experienced by more than one-third of the participants, and the highest self-rated negative impact was linked to increased or novel symptoms, as well as lack of quality in the treatment and therapeutic relationship.

## Availability

The NEQ is freely available for use in research and clinical practice At time of writing, the instrument has been translated by professional translators into the following languages, available for download via the website www.neqscale.com: Danish, Dutch, English, Finnish, French, German, Italian, Japanese, Norwegian, Spanish, and Swedish.
